# 

**Published:** 1853-01

**Authors:** 


### TO READERS AND CORRESPONDENTS,

Communications intended for publication, and all letters relating to the business of the Journal, or containing remittances in money, should be directed to Drs. Harris and Blandy, Baltimore, Md.

### *Medical and Dental Journals Received.*

1. *The American Journal of the Medical Sciences.* Edited by Isaac Hays, M. D., Surgeon to Wills Hospital, &c., October, 1852. *In Exchange.*

2. *The New York Dental Recorder.* Edited by C. C. Allen, M. D., Dentist. July, October, November and December, 1852. *In Exchange.*

3. *The Dental Register of the West.* Edited by James Taylor, M. D., D. D. S. Published by order of the Mississippi Valley Association of Dental Surgeons. October, 1852. *In Exchangc.*

4. *The Dental News Letter.* October, 1852. *In Exchange.*

5. *The London Medical Gazette.* August and September, 1852. *In Exehange.*

6. *Boston Medical and Surgical Journal.* Edited by J. V. C. Smith, M. D. July, November and December, 1852. *In Exchange.*

7. *The British and Foreign Medico-Chirurgical Review.* October, 1852. *In Exchange.*

8. *The Ohio Medical and Surgical Journal.* Edited by Richard L. Howard, M. D. October, November and December, 1852. *In Exchange.*

9. *The New York Journal of Medicine and the Collateral Sciences.* Edited by S. S. Purple, M. D. November and December, 1852. *In Exchange.*

10. *The Medical Examiner.* Edited by F. G. Smith, M. D., and John Biddle, M. D. October, November and December, 1852. *In Exchange.*

11. *The Southern Medical and Surgical Journal.* Edited by J. P. Garvin, M. D. October, November and December, 1852. *In Exchange.*

12. *The New York Medical Gazette, and Journal of Health.* Edited by D. M. Reese, M. D., LLD. October, November and December, 1852. *In Exchange.*

13. *The New Jersey Medical Reporter, and Transactions of the New Jersey Medical Society.* Edited by JOSEPH PARISH, M. D. October, November and December, 1852. *In Exchange.*

14 *The Western Lancet and Hospital Reporter.* Edited by L. M. LAWSON, M. D., and GEORGE MENDENHALL, M. D. Not received *In Exchange.*

15. *The North Western Medical and Surgical Journal.* Edited by J. EVANS, M. D., and E. G. MEEK, A. M., M. D. October, November and December, 1852. *In Exchange.*

16. *The Western Journal of Medicine and Surgery.* Edited by L. P. YANDELL, M. D., and T. S. BELL, M. D. October, November and December, 1852. *In Exchange.*

17. *The New Hampshire Journal of Medicine.* Edited by EDWARD H. PARKER, A. M., M. D. October, November and December, 1852. *In Exchange.*

18. *The Charleston Medical Journal aud Review.* Edited by D. J. CAIN, M. D., and F. PEYREL PORCHER, M. D. October and November, 1852. *In Exchange.*

19. *The Buffalo Medical Journal.* Edited by AUSTIN FLINT, M. D. October, November and December, 1852. *In Exchange.*

20. *The Provincial Medical aud Surgical Journal.* Edited by G. W. H. RANKIN, M. D., and J. H. WALSH, Esq. September, October and November, 1852. *In Exchange.*

21. *The London Lancet*, Journal of British and Foreign, Surgical and Chemical Sciences, Chemistry, Literature and News. Editor THOMAS WAKELEY, M. D., for London. Sub-Editors, J. H. BENNET, M. D., and T. WAKELEY, M. R. C. S. E. October and November, 1852. *In Exchange.*

22 *The Northern Lancet and Gazette of Legal Medicine.* A monthly Journal of Medicine and General Science, Criticism, and Medical Jurisprudence. Edited by HORACE NELSON, M. D., and FRANCIS J. D. AVIGNON, M. D. October, November and December, 1852. *In Exchange.*

23. *The Dublin Medical Press.* October, November and December, 1852. *In Exchange.*

